# Problematic Purpura

**DOI:** 10.7759/cureus.13473

**Published:** 2021-02-21

**Authors:** Marina K Ibraheim, Sheela Gavvala

**Affiliations:** 1 Dermatology, John P. and Kathrine G. McGovern School of Medicine, University of Texas Health Science Center, Houston, USA; 2 Pediatrics, John P. and Kathrine G. McGovern School of Medicine, University of Texas Health Science Center, Houston, USA

**Keywords:** pediatrics, purpura, post-emesis, petechiae

## Abstract

Post-emetic purpura is an often-forgotten cause of facial rash in the acute setting and can often be mistaken for more dangerous etiologies. We present a case of a child recently treated with trimethoprim/sulfamethoxazole for impetigo who demonstrated a new rash after multiple episodes of vomiting. Lab workup, in conjunction with patient history and lesion location, pointed toward a diagnosis of post-emetic purpura. Careful consideration of the history, physical examination findings, location of the lesion, and laboratory findings are essential for diagnosing post-emetic purpura and differentiating it from other etiologies acutely.

## Introduction

Petechia, purpura, and ecchymoses are nonblanching lesions that occur due to extravasation of blood into the skin [1,2]. In the acute setting, the presence of petechia and purpura can prove alarming, as many life-threatening etiologies can present in this manner [3]. We present a case of a child recently treated with trimethoprim/sulfamethoxazole for impetigo who demonstrated a new rash after multiple episodes of vomiting. Post-emetic purpura must be considered as part of a differential diagnosis for facial purpura. 

## Case presentation

**Figure 1:**
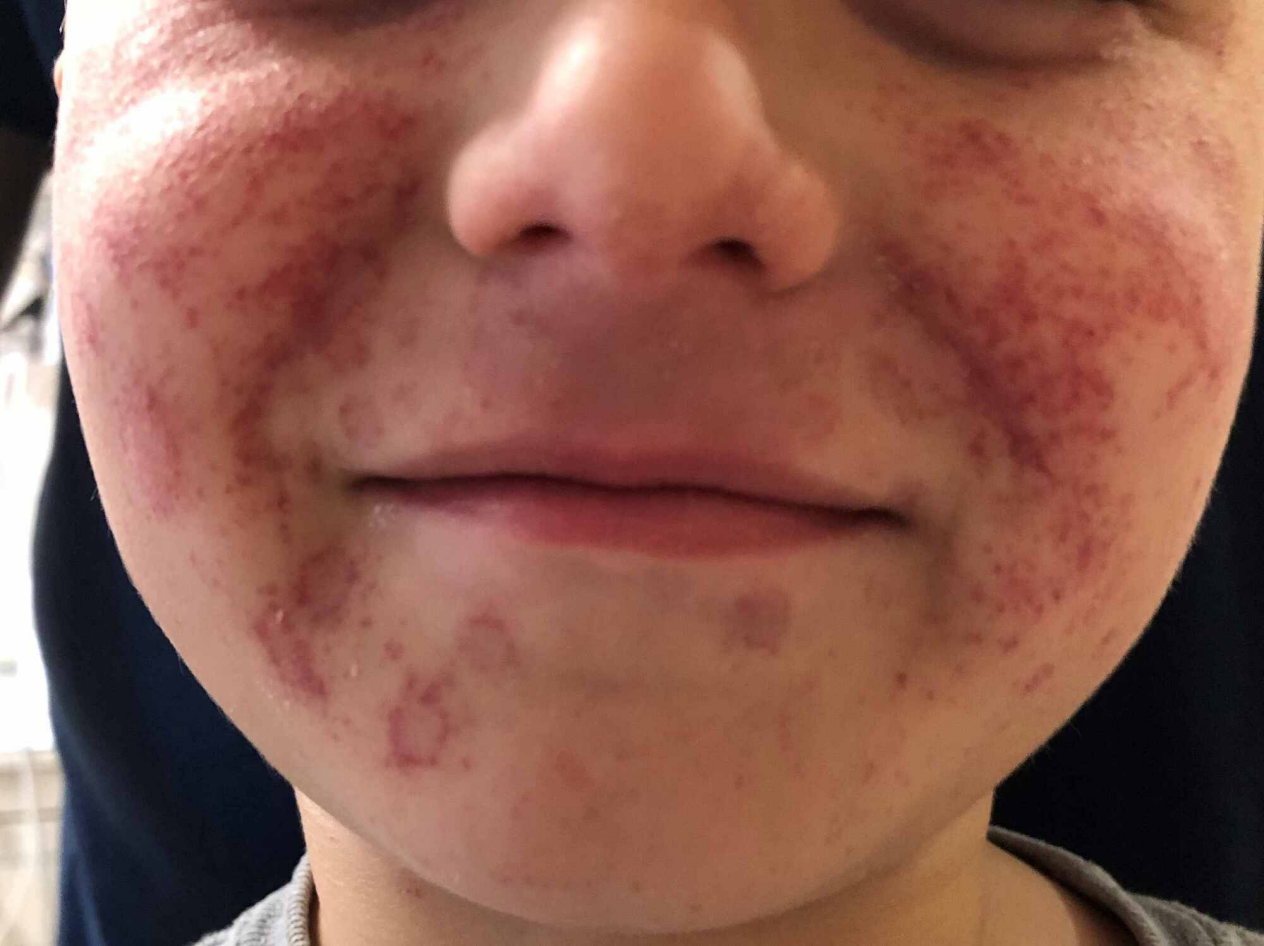
Diffuse micropurpura on the face Nonblanching erythematous, purpuric macules scattered across his cheeks, chin, and between his eyebrows. The same lesions were visible above his eyelids.

**Figure 2:**
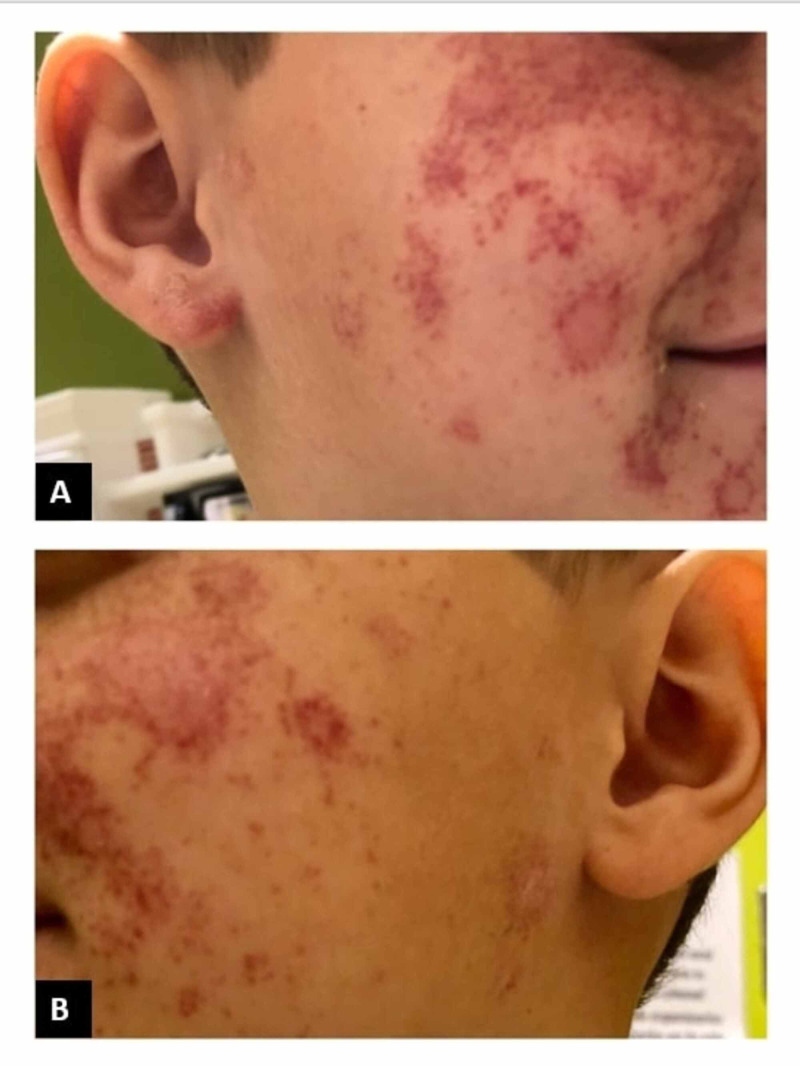
Purpuric lesions on the right and left cheeks (A) Nonblanching, erythematous, purpuric macules, some coalescing into rings with irregular borders on the right cheek. (B) Similar lesions on the left cheek.

A five-year-old boy with a recent history of impetigo presented to the hospital with a facial rash. Two weeks prior, he received trimethoprim/sulfamethoxazole for treatment of disseminated impetigo that affected his face, trunk, and genitalia. Hours before admission, the patient vomited 10 times and, after the third episode of vomiting transpired, developed a new, impressive facial rash, abdominal pain, and diarrhea. On admission, he was afebrile and normotensive. Physical examination demonstrated countless nonblanching erythematous purpuric macules scattered across his cheeks, chin, between his eyebrows, and above his eyelids (Figures 1, 2).

Laboratory testing of the complete blood count revealed leukocytosis to 19.7x10^3^ k/cmm; the hemoglobin and platelet count were 11.7 g/dL and 308x10^3^ k/cmm, respectively, which were within normal limits. Bleeding time, prothrombin time, and partial thromboplastin time were not obtained. Abdominal x-ray did not reveal any acute abnormalities. The patient was admitted to the floor, where he was monitored and given IV fluids. During admission, he remained afebrile and normotensive; no episodes of diarrhea or vomiting occurred. Based on history, physical examination, and laboratory findings, the team diagnosed him with post-emetic purpura in the setting of gastroenteritis. After 24 hours, the patient was discharged.

## Discussion

Petechia, purpura, and ecchymosis refer to nonblanching lesions that manifest after blood has extravasated from the vasculature and into skin [1]. Petechia is less than 2 mm in diameter, whereas purpura is between 2 and 10 mm; ecchymoses are greater than 10 mm in size [2]. In the acute setting, the presence of petechia and purpura can prove alarming, as many life-threatening etiologies can present in this manner: vasculitides, infections, neoplasias, coagulopathies, drug eruption, and connective tissue disease [3,4]. Another etiology to consider in the pediatric population is post-emetic purpura [5,6].

Post-emetic purpura occurs due to an increase in intrathoracic pressure; vomiting, Valsava, coughing, crying, delivery, and endoscopy have been described to result in this phenomenon [2-8]. Distinguishing this cause of purpura from other causes relies upon identification of the location of the lesion. Post-emetic purpura classically manifests on the face, especially in the periorbital area. This area is highly vascularized, resulting in a greater propensity for blood extravasation in response to elevated pressure. In contrast, vasculitides such as Henoch-Schonlein purpura typically manifests on the legs of children with gastrointestinal symptoms [2]. Consideration of the clinical picture in conjunction with lab testing can rule out various etiologies; derangements in the complete blood count and coagulation studies can highlight coagulopathies, thrombocytopenia, platelet derangements, and infections [1]. Urinalysis and fecal occult blood studies can be performed to rule out vasculitides. Notably, the urinalysis in our case demonstrated ketonuria, which would be consistent with dehydration secondary to repeated vomiting. If all other diagnoses have been ruled out and the purpura exists solely on the face, then post-emetic purpura ought to be suspected; no skin biopsy is required to diagnose this [2].

Treatment of post-emetic purpura is supportive, as these lesions are not bothersome and will resolve after several days [2,5].

## Conclusions

Post-emetic purpura remains an often-forgotten cause of facial rash in the acute setting and should be kept as part of the differential for facial purpura. Careful consideration of the history, physical examination findings, location of the lesion, and lab studies can aid in diagnosis of this condition and in ruling out other etiologies that must also be considered in the acute setting.
